# Detection and characterisation of sheep-associated malignant catarrhal fever infection from ruminants by using tegument and gB gene sequences of OvHV-2

**DOI:** 10.4102/ojvr.v87i1.1886

**Published:** 2020-11-11

**Authors:** Tuba Ç. Oğuzoğlu, Seçkin Salar, Ece Adıgüzel, Cansu Demirden, Onur Ülgenalp

**Affiliations:** 1 Department of Virology, Faculty of Veterinary Medicine, Ankara University, Ankara, Turkey; 2 Department of Obstetric and Gnynecology, Faculty of Veterinary Medicine, Ankara University, Ankara, Turkey

**Keywords:** malignant catarrhal fever, tegument gene, gB gene, OvHV-2, Turkey

## Abstract

In this study, positive blood and organ samples were obtained from different mixed herds of sheep and cattle against ovine herpesvirus 2 (OvHV-2) infection. Target-positive DNA was sequenced and compared with worldwide distributed OvHV-2 sequences. Tegument gene (422 base pairs) and glycoprotein B (gB) gene (2800 base pairs) amplicons of OvHV-2 genome were used for understanding of epidemiology of malignant catarrhal fever (MCF) infection in Turkey. The results of nucleotide sequencing of polymerase chain reaction (PCR) products indicated presence of sheep-associated form for MCF infection in Turkey. Although the obtained sequences were genetically different from each other, it was found that genetic variations were limited.

## Introduction

Known as a fatal infection, malignant catarrhal fever (MCF) affects susceptible cattle, wild ruminants and pigs (Plowright, Ferris & Scott 1960). Two causative agents for MCF are predominantly described, one of them carried by sheep infected with ovine herpesvirus 2 (OvHV-2) (called sheep-associated MCF) and the other carried by wildebeest (*Connochaetes* species) containing alcelaphine herpesvirus 1 (AlHV-1) (called wildebeest-associated form) (Roizman 1992; Russel, Stewart & Haig 2009). To date, more than 10 identified causative viruses have been associated with this infection in domestic and wildlife ruminants (Crawford et al. 2002; Nthiwa et al. 2019; Seeley et al. 2018).

The clinicopathological findings of MCF in affected animals are not clear about differential diagnoses. Recently, new forms, neurological syndrome and systemic necrotising vasculitis, have been described that make determining clinical pictures and diagnosing the disease difficult (Martins et al. 2017; Pesavento et al. 2019). Famous findings have described high fever, generalised lymphadenopathy, keratoconjunctivitis, corneal oedema and opacity, but these symptoms are not always evident (Plowright 1990).

The tegument gene OvHV-2 is encoded by open reading frame (ORF) 33 and ORF 75 and used for the purpose of detection of OvHV-2 DNA and phylogenetic analysis based on sequence data (Baxter et al. 1993). The glycoprotein B (gB) gene of OvHV-2 is the most conserved herpes virus protein, encoded by ORF 8 (Pereira 1994). Glycoprotein B plays a role in virus entry to a host organism and spread between cells. By using this on primers of the gB gene, it is possible to investigate the geographical localisation amongst the sequence variations of OvHV-2 strains (Dunowska et al. 2001) or evaluate vaccine targets for AlHV-1/OvHV-2 chimeric viruses (Cunha et al. 2016).

The present report describes the detection of an MCF infection circulating in cattle and sheep in Turkey. A molecular comparison between cattle and sheep strains was made using the tegument and gB gene sequences of OvHV-2 to explain the origin of the infection.

## Materials and methods

**FIGURE 1:**
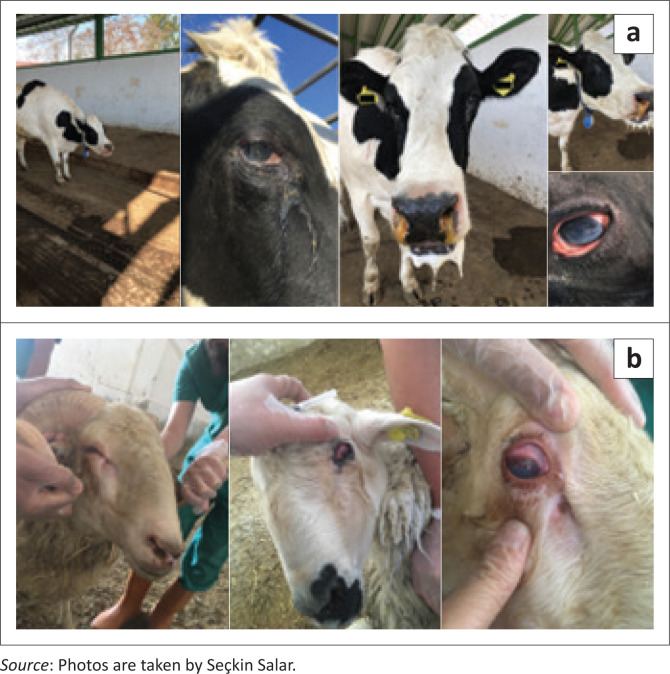
(a) Clinical signs of an affected cow; (b) clinical signs of affected sheep.

In total, eight animals from three different farms were sampled in this study. Whole-blood sample was taken from dairy cattle with clinical symptoms. Additionally, blood samples were taken from five sheep located close to the cattle boxes at the same farm. One of 50 lactating cows that contracted the disease showed the main clinical symptoms of persistent high fever (> 41 °C), dyspnea, nasal and ocular discharges, conjunctival and scleral hyperemia and keratoconjunctivitis with corneal opacity (Figure 1a). The cow was separated 5 days after the first symptoms of the disease were detected. Five of 145 sheep housed in the same barn showed signs of high fever (> 40.5 °C), scleral hyperemia and nasal and ocular discharges, and they were separated from the flock (Figure 1b).

Additionally, a cow and a sheep from different farms in the same village were sampled at different time intervals. At the farm where the cow was sampled, there were 80 sheep, 100 yearlings and 32 cattle. For this farm, a veterinarian reported that six cattle had died. In addition, sheep deaths were reported from a sheep-only farm in the same village, so a lung sample was taken from a dead sheep by the veterinarian and sent to the laboratory for diagnosis.

Viral DNA was extracted from sampling materials using the Phenol:Chloroform:Isoamlyalchohol (25:24:1) method described by Sambrook, Fritsch and Maniatis (1989). For detection of infection, polymerase chain reactions (PCRs) were performed using the primers 556/775/555 that code the tegument gene of OvHV-2 as previously described by Baxter et al. (1993) for hemi-nested PCR. The first round yielded a 422-base-pair (bp) PCR product, and the nested round yielded a 238-bp PCR product. Secondly, the ORF 8 primer pair (91/116) was used to amplify the OvHV-2 gB gene described by Dunowska et al. (2001) for molecular characterisation. The PCR amplifications were modified for annealing temperatures (55 °C for 556/775/555 primers, 50 °C for 91/116 primers). Polymerase chain reaction products were visualised by agarose gel electrophoresis. Positive amplicons were purified and sequenced commercially.

Phylogenetic analyses were performed using the BioEdit (Hall 1999) and MEGA (Tamura et al. 2013) software programmes. The percentages of replicate trees in which the associated taxa clustered together in the bootstrap test (1000 replicates) are shown next to the branches. The evolutionary distances were computed using the Kimura 2-parameter method and are in the units of the number of base substitutions per site.

### Ethical consideration

The authors confirm that ethical clearance was not required for the study.

## Results

**FIGURE 2:**
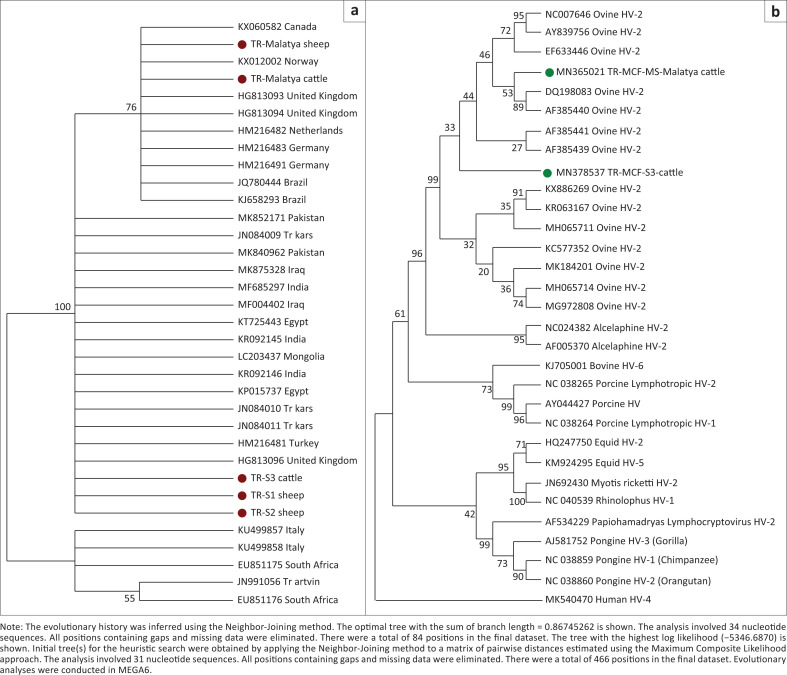
Phylogenetic analyses by using tegument (a) and gB (b) gene sequences of ovine herpesvirus 2 strains in this study.

One cow from the first farm was found positive for MCF by using the tegument gene primers of OvHV-2. Cattle and sheep were being kept together in this farm. Additionally, in two blood samples from five sheep obtained from the same farm, DNA of OvHV-2 was found. All three positive sequences for the tegument gene were closely related to each other (Figure 2a). This result indicates the presence of an infection transferring to cattle by contact with infected sheep.

The tegument gene sequences of samples from a sheep and a cow taken from the different farms in the same region were very close to each other, just as in the first farm. It was concluded that the infection in this village occurred as a result of transmission by contact between nearby farms.

All five tegument gene sequences in this study were closely related to each other. Whilst gB gene primers reacted only with bovine samples, tegument gene primers reacted in both ovine and bovine samples.

Bovine-originated OvHV-2 viruses in this study were in separate phylogenetic branches as they came from different geographical areas, yet both of them were affected by the group of ovine-derived herpes viruses (Figure 2b).

The amplicons obtained from positive samples for the gB gene of OvHV-2 were ~2800 bp. Comparison of deduced amino acid sequences from gB gene between our MCF strains and the reference strains obtained from GenBank has shown that the S3 strain had more amino acid substitution changes than the *Malatya* sample (MS) (data not shown). Comparing the gB gene sequences of Turkish MCF strains, the identity rates were estimated at 99.3% by using Clustal W algorithm (data not shown). The similarity rates between Turkish MCF strains and other reference strains (DQ198083, AY839756, KC577352, KR063167, AF385441, MH065713) from GenBank ranged from 98.6% to 99.1% (data not shown). Similarly, the divergence percentage between two Turkish MCF strains was found to be 0.7%; and with other strains from GenBank, it was found to range from 0.9% to 1.4%.

## Discussion

Serological (Yeşilbağ 2007) and virological studies (Yazıcı et al. 2006; Yildirim et al. 2012) generally report the presence and surveying of MCF infections in the asymptomatic carrier and affected animals in Turkey. The first goal of this study was to detect the DNA of OvHV-2 in diagnostic samples from suspected animals infected with MCF. The second goal, the purpose of determining the origin of the virus, was to make a comparison between cattle and sheep OvHV-2 sequences obtained from affected animals.

In this study, we compared the tegument gene and gB gene sequences of OvHV-2 viruses (Figures 1 and 2), which were obtained from cattle and sheep from two different geographical regions in Turkey. The first positive results belonged to the samples taken from one cow and five sheep in the same farm, and they all presented suspicious clinical findings related to MCF. The cow and the two sheep were found to be positive for OvHV-2 in PCR. The other positive results were acquired from a single sheep and a single cow that were sampled at different time intervals from different farms. Our results show that the viruses obtained from the same regions or the same farm match each other, and the disease could be identified as sheep-associated MCF. According to the phylogenetic analysis of the tegument gene, MSs that were taken from a cow and a sheep in the same village but different farms were closely related to the OvHV-2 sequences obtained from Canada, Norway and UK samples. They were also very similar to each other. The other three tegument-gene-positive samples in this study belonged to one cow and two sheep in the same farm. They were also quite close to each other. Interestingly, in the phylogenetic tree, these samples were located closer to other tegument gene sequences (from Kars) obtained from another study in Turkey (Yildirim et al. 2012) than other samples in the current study. Generally, the genetic identity amongst Turkish MCF samples and from those of other parts of the world has seen no significant genomic variations.

We were able to compare the tegument gene sequences because the amplicons were obtained by using the mentioned gene primers. Interestingly, only positive samples that were bovine originated could be evaluated in terms of gB genes, whilst sheep-originated samples did not react with gB gene primers. It is concluded that this situation is important for laboratory diagnosis; hence, we recommend using the tegument gene instead of the gB gene in PCR for MCF diagnostics.

Malignant catarrhal fever is a fatal disease for cattle, the last hosts of this infection. Although experimental vaccine studies have been reported for cattle (Haig et al. 2008; Lankester et al. 2016; Russel et al. 2012), there is no commercial product available in common use because of an insufficient antibody response in terms of intensity and duration. Prevention of MCF has classically focused on control of transmission of OvHV-2 between sheep and cattle. By separating sheep from susceptible species like cattle, the infection can be limited (Li et al. 1999). Although there is known to be a sheep-originated MCF infection in Turkey, there is a necessity to investigate different viruses that can be obtained from different animal species (sheep, goats, cattle and wild ruminants). Thus, we believe that the acquisition of viruses that are adapted to cell culture, which are potential vaccine candidates, will allow us to fight MCF infections.

In conclusion, OHV-2 tegument gene sequences obtained from cattle and sheep in this study were very closely related. This finding supports the hypothesis that subclinically infected sheep are the source of the infectious virus for susceptible dairy cattle. Although limitation of sheep and cattle rearing together is important to prevent this infection, we believe that prophylactic protection measures may be more preferable in mixed herds. In this context, we believe that there will be a need for field isolates to be adapted to cell culture and that studies should be conducted in this direction.
